# Recent Advances in Two-Dimensional Spintronics

**DOI:** 10.1186/s11671-020-03458-y

**Published:** 2020-12-09

**Authors:** Guojing Hu, Bin Xiang

**Affiliations:** 1 Department of Materials Science and Engineering, CAS Key Lab of Materials for Energy Conversion, Hefei National Research Center for Physical Sciences at the Microscale, University of Science and Technology of China, Hefei, 230026 Anhui China; 2 Anhui Laboratory of Advanced Photon Science and Technology, University of Science and Technology of China, Hefei, 230026 China

**Keywords:** 2D spintronics, Graphene, Topological insulator, Van der Waals magnet, Spin-charge conversion, Spin transport, Spin manipulation

## Abstract

Spintronics is the most promising technology to develop alternative multi-functional, high-speed, low-energy electronic devices. Due to their unusual physical characteristics, emerging two-dimensional (2D) materials provide a new platform for exploring novel spintronic devices. Recently, 2D spintronics has made great progress in both theoretical and experimental researches. Here, the progress of 2D spintronics has been reviewed. In the last, the current challenges and future opportunities have been pointed out in this field.

## Introduction

With the discovery and application of the giant magnetoresistance effect (GMR), spintronics has quickly been developed into an attractive field, aiming to use the spin degree freedom of electrons as an information carrier to achieve data storage and logical operations [1–3]. Compared to conventional microelectronic devices based on charge, spintronic devices require less energy to switch a spin state, which can result in faster operation speed and lower energy consumption. Therefore, spintronics is the most promising technology to develop alternative multi-functional, high-speed, low-energy electronic devices. Although spin-transfer-torque magnetoresistive random-access memory (STT-MRAM) has been commercially produced, various technical issues still need to be resolved. Major challenges include the efficient generation and injection of spin-polarized carriers, long-range transmission of spin, and manipulation and detection of spin direction [4–6].

In parallel with the boom of spintronics, two-dimensional (2D) van der Waals (vdW) materials have been at the frontier of material research since the isolation of graphene [7–9]. Distinct from their bulk materials, 2D vdW materials exhibit many novel physical phenomena. Some 2D materials have already shown great potential for the engineering of next-generation 2D spintronic devices [10–12]. For example, graphene exhibits high electron/hole mobility, long spin lifetimes, and long diffusion lengths, which make it a promising candidate for a spin channel [13–15]. However, due to its characteristics of zero gap and weak spin–orbit coupling (SOC), graphene has limitations in building graphene-based current switches. In contrast, 2D transition metal dichalcogenides (TMDCs) have varied band gaps, strong SOC effect, and, especially, unique spin-valley coupling, providing a platform to manipulate spin and valley degrees of freedom for nonvolatile information storage [16, 17]. Topological insulators (TIs) with topologically protected surface states have strong spin–orbit interactions to achieve spin-momentum locking, which can suppress scattering and enhance spin and charge conversion efficiency [4, 12, 18]. Emerging 2D magnets with intrinsic magnetic ground states down to atomic-layer thicknesses open up new avenues for novel 2D spintronic applications [19–21].

With the development of 2D spintronics, it is necessary to review the latest experimental and theoretical work in the field. In this article, the progress of 2D spintronics has been reviewed, and some current challenges and future opportunities have also been discussed in this emerging field. The first section reviews magnetism in 2D materials, including induced magnetic moments in graphene, TIs, and some other 2D materials via the methods of doping or proximity effect, and some intrinsic 2D magnets. The second section presents the three elementary functionalities to achieve 2D spintronic device operations, including spin-charge conversion, spin transport, and spin manipulation in 2D materials and at their interfaces. The third section overviews applications of 2D spintronics. The fourth section introduces several potential 2D spintronic devices for memory storage and logic applications. The final section discusses some current challenges and future opportunities in 2D spintronics to achieve practical application.

## Magnetism in 2D Materials

Magnetism has important meanings in data storage technologies. However, most 2D materials like graphene are not intrinsically magnetic. Two methods have been proposed to make nonmagnetic materials magnetic. The first method is to generate spin polarization by introducing vacancies or adding adatoms [22–24]. The other one is to introduce magnetism via the magnetic proximity effect with the adjacent magnetic materials [18, 25, 26]. The recently discovered 2D magnetic vdW crystals have intrinsic magnetic ground states at the atomic scale, providing unprecedented opportunities in the field of spintronics [20, 27].

### Induced Magnetic Moments in Graphene

**Fig. 1:**
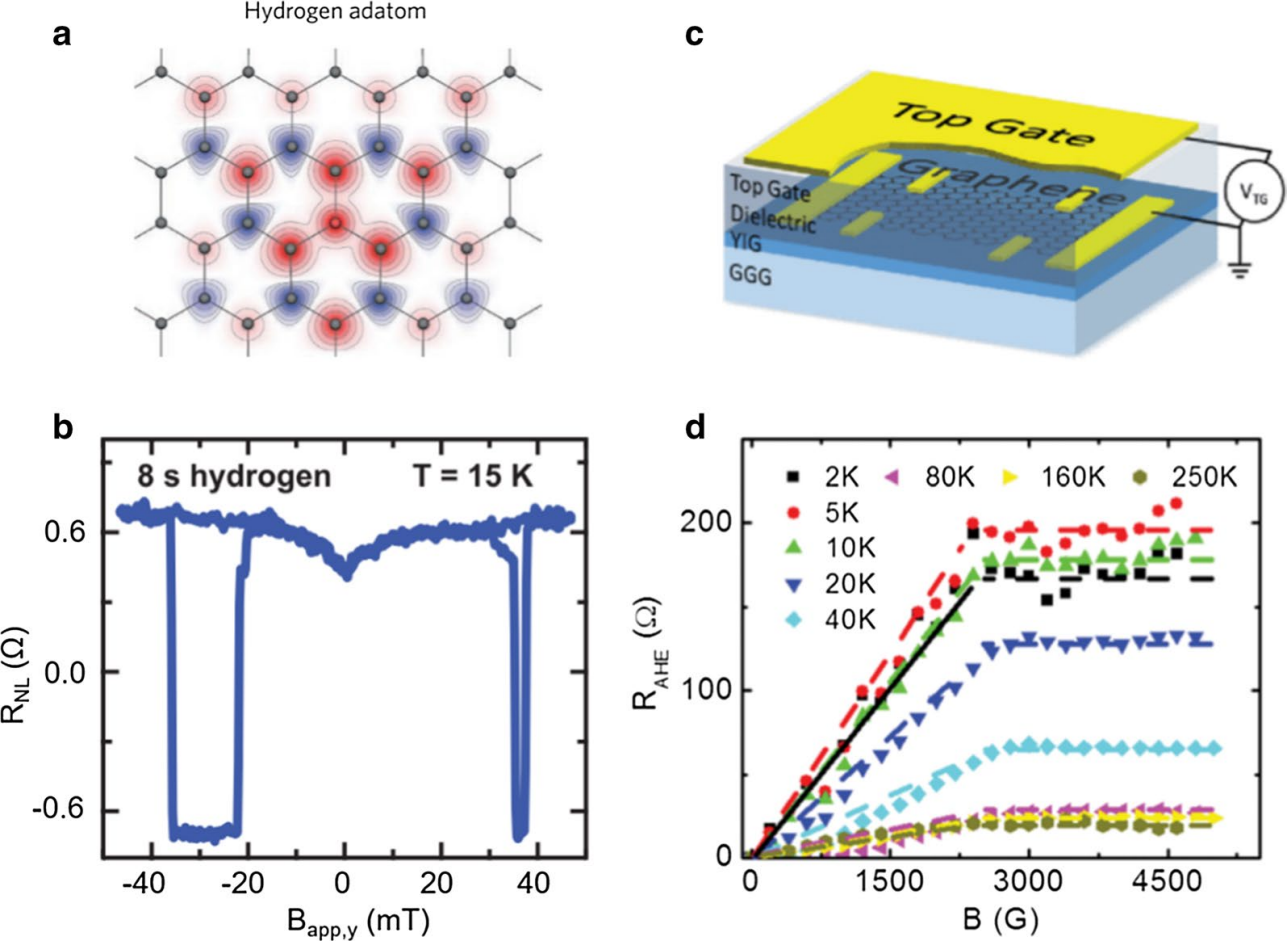
Induced magnetic moment in graphene. **a** Theoretical prediction of magnetic moments in graphene due to hydrogen. **b** Magnetic moments due to hydrogen doping detected by spin transport measurements at 15 K. The device was measured after 8 s hydrogen doping. **c** Schematic of graphene exchange coupled to an atomically flat yttrium iron garnet (YIG) ferromagnetic thin film. **d** Anomalous Hall resistance measurements on magnetic graphene at different temperatures. **a**, **b** Reproduced with permission from McCreary et al., Phys. Rev. Lett. 109, 186,604 (2012). Copyright 2012 American Chemical Society [23]. (c) and (d) reproduced with permission from Wang et al., Phys. Rev. Lett. 114, 016,603 (2015). Copyright 2015 American Chemical Society [25]

Pristine graphene is strongly diamagnetic, so a large number of theoretical and experimental studies explore the magnetism of graphene. Introducing vacancies and adding hydrogen or fluorine have been used to induce magnetic moments in graphene [23, 25, 28]. For example, Kawakami’s group utilized hydrogen adatoms to dope the graphene (Fig. 1a) and detected pure spin current by nonlocal spin transport measurement to demonstrate magnetic moment formation in graphene [23]. As shown in Fig. 1b, the characteristic dip appearing at zero magnetic field in the nonlocal spin transport measurement shows that the pure spin current is scattered by exchange coupling between conduction electrons and local hydrogen-induced magnetic moments. In addition, graphene with fluorine adatoms and vacancy defects has paramagnetic moments, which can be measured by a SQUID (superconducting quantum interference device) [28]. Nevertheless, the realization of long-range ferromagnetic order in doped graphene is still an overwhelming challenge. Some researchers have proposed using the magnetic proximity effect to make graphene gain magnetism [29]. When graphene is adjacent to a magnetic insulator, the π orbitals of graphene and the neighboring spin-polarized d orbitals in the magnetic insulator have an exchange interaction to generate long-range ferromagnetic coupling. As shown in Fig. 1c, in the graphene/yttrium iron garnet (YIG) heterostructure, the measured anomalous Hall effect signal can persist to 250 K (Fig. 1d) [25].

### Induced Magnetic Moments in TIs

**Fig. 2:**
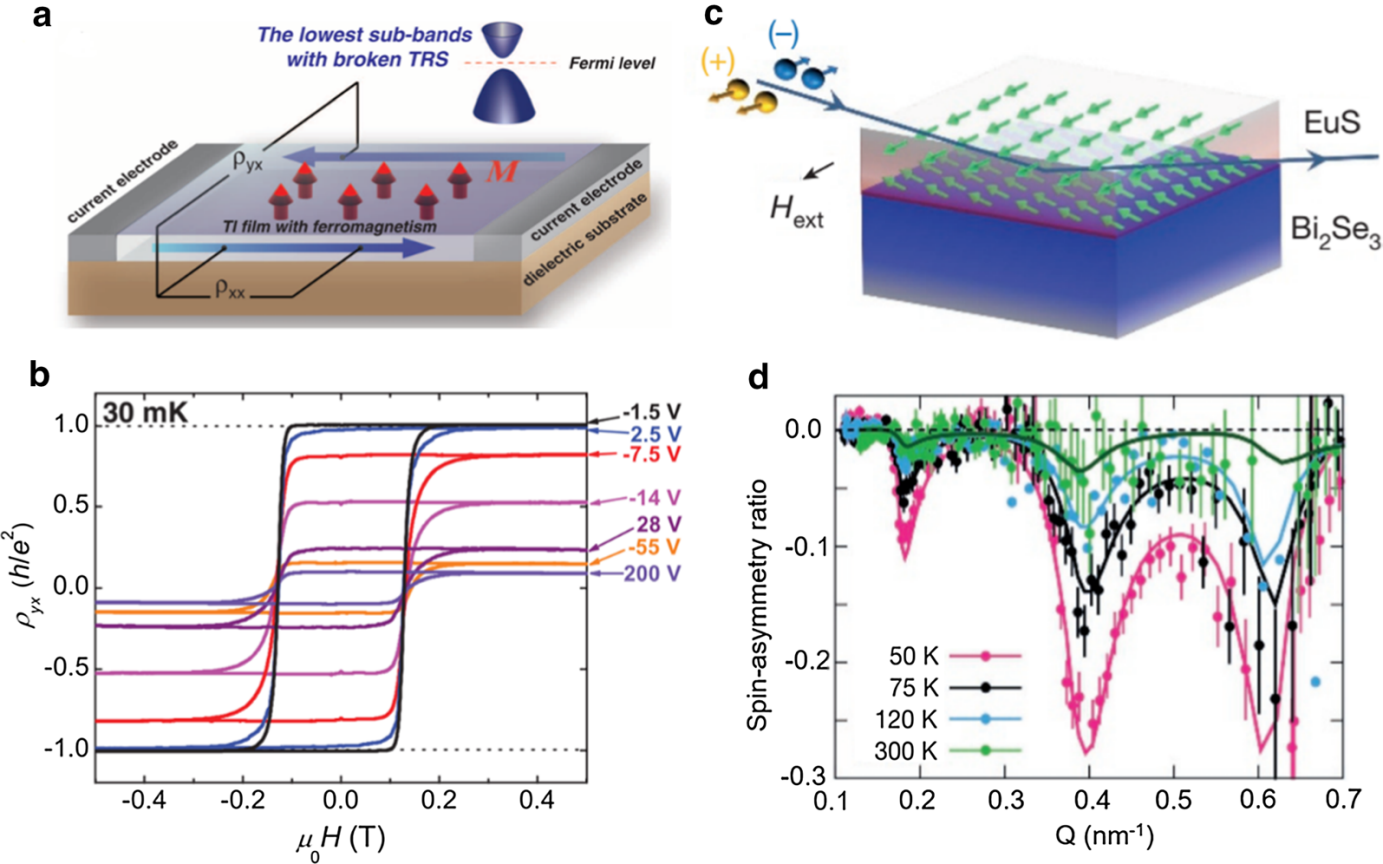
Induced magnetic moment in TIs. **a** Schematic of the QAHE in a magnetic TI thin film. The magnetization direction (M) is indicated by red arrows. The chemical potential of the film can be controlled by a gate voltage applied on the back side of the dielectric substrate. **b** Magnetic field dependence of QAHE at different gate voltages in Cr_0.15_(Bi_0.1_Sb_0.9_)_1.85_Te_3_ film. **c** Schematic of the polarized neutron reflectivity (PNR) experiment for Bi_2_Se_3_/EuS bilayer film. **d** Observation of ferromagnetic order in Bi_2_Se_3_/EuS bilayer sample via magnetic proximity coupling to the EuS measured by PNR measurements. **a**, **b** Reproduced with permission from Chang et al., Science 340, 167 (2013). Copyright 2013 The American Association for the Advancement of Science [24]. **c, d** Reproduced with permission from Katmis et al., Nature 553, 513 (2016). Copyright 2016 Nature Publishing Group [32]

2D materials are susceptible to environmental conditions, such as moisture and oxygen. The conductive surface state in TI surface regions is considered to be a more stable 2D material [30]. In addition, the surface state of TIs exhibits the spin-momentum locking property, which provides a way to manipulate the spin signal via the charge current direction. More interestingly, breaking the time-reversal symmetry by the doping of magnetic atoms or the magnetic proximity effect can give rise to some exotic phenomena such as the quantum anomalous Hall effect (QAHE) [18, 31]. Chang et al. [24] first observed QAHE in Cr doped magnetic TI, Cr_0.15_(Bi_0.1_Sb_0.9_)_1.85_Te_3_. As demonstrated in Fig. 2a, by tuning the Fermi level of magnetically induced TI bands, we can observe a plateau of Hall conductance of *e*^2^/*h*. The measured results show the gate-tunable anomalous Hall resistance reaches the quantized value of *h*/*e*^2^ at zero magnetic field (Fig. 2b). However, the spin scattering effect of doped magnetic atoms is limited to achieve a robust long-range magnetic order at the surface of the TI. The magnetic proximity between TIs and magnetic materials can avoid the introduction of doping atoms or defects, gaining a long-range magnetic order by interfacial exchange coupling. Spin-polarized neutron reflectivity (PNR) was utilized to study the interface magnetism at the heterostructure of Bi_2_Se_3_/EuS (Fig. 2c) [32]. The PNR result shows that the Bi_2_Se_3_/EuS bilayer has a ferromagnetic order at the interface, and this topologically enhanced interfacial ferromagnetism can persist up to room temperature (Fig. 2d). Realizing a ferromagnetic surface state in a TI is predicted to allow several prominent phenomena to emerge, such as the interfacial magnetoelectric effect [33] and the electric field-induced image magnetic monopole [34].

### Induced Magnetism in Other 2D Materials

Besides graphene and TIs, magnetism induced by intrinsic defects and dopants in other 2D materials have also been investigated, including phosphorene [35], silicene [36, 37], GaSe [38], GaN [39], ZnO [40], etc. First-principles calculation results showed that an interplay between vacancy and external strain can give rise to magnetism in phosphorene. When a strain is along the zigzag direction of phosphorene and P vacancies reaches 4%, the system exhibits a spin-polarized state with a magnetic moment of ~ 1 *μ*_B_ per vacancy site [35]. First-principles calculations also predicted that hole doping can induce ferromagnetic phase transition in GaSe and GaS, due to exchange splitting of electronic states at the top of the valence band. The magnetic moment can be as large as 1.0 *μ*_B_ per carrier [38, 39]. However, most of these investigations are limited to theoretical calculations. Further studies, particularly experimental work are needed to understand the magnetic behaviors and to explore robust 2D room temperature ferromagnetic semiconductor for practical applications.

### Intrinsic 2D Magnets

**Fig. 3:**
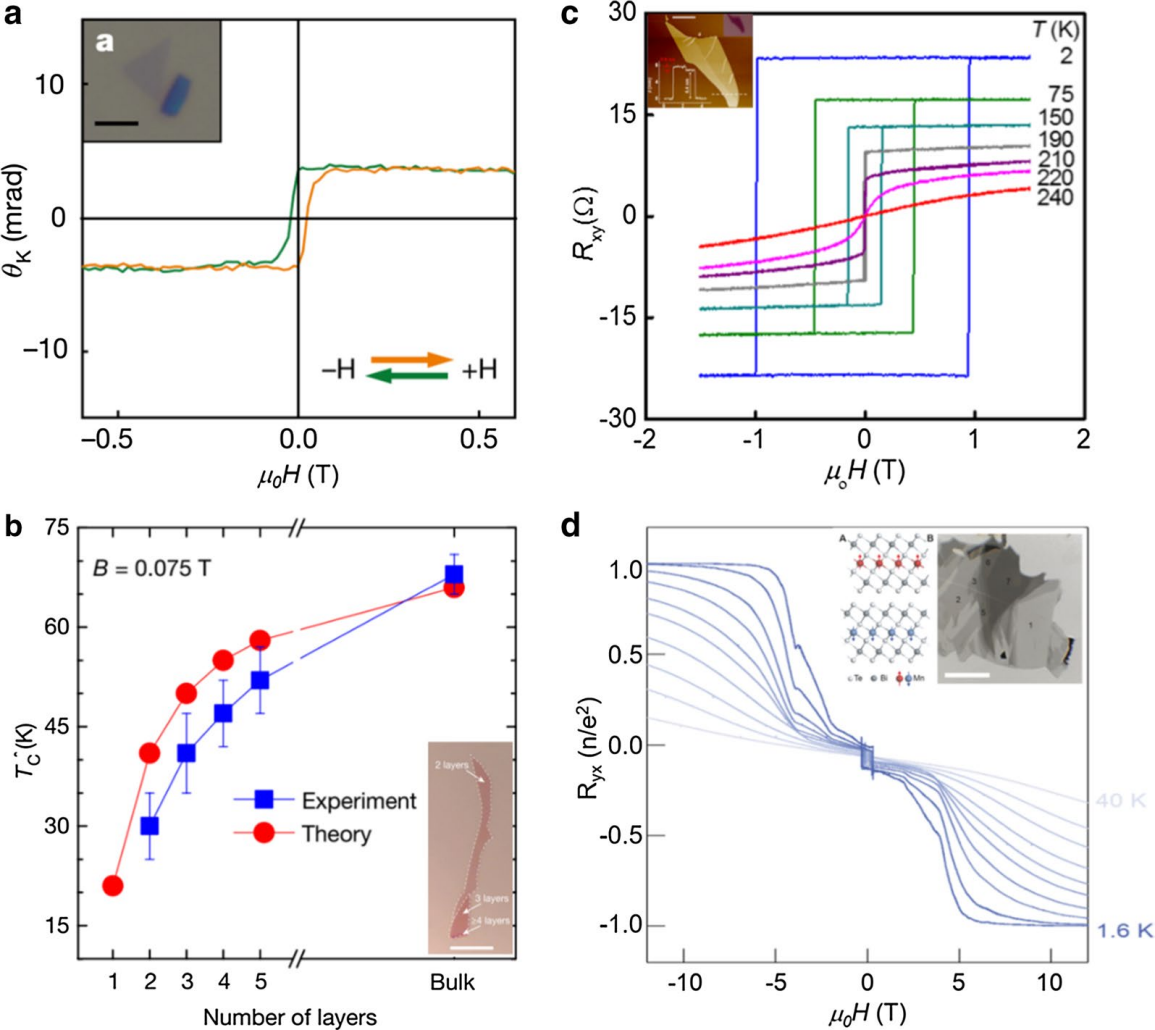
Intrinsic 2D magnets. **a** Polar magneto-optical Kerr effect (MOKE) signal for a CrI_3_ monolayer. The inset shows an optical image of an isolated monolayer CrI_3_. **b** Transition temperatures T_C∗_ of Cr_2_Ge_2_Te_6_ for different thicknesses, the plot with blue squares obtained from Kerr measurements, and the plot with red circles from theoretical calculations. The inset shows an optical image of exfoliated Cr_2_Ge_2_Te_6_ atomic layers on SiO_2_/Si. **c** Temperature-dependent magnetic field sweeps of the Hall resistance measured on a 12-nm-thick Fe_3_GeTe_2_ device. The inset shows an atomic force microscope image of a representative thin FGT flake on SiO_2_. **d** Magnetic field-induced QAHE in a five-layer MnBi_2_Te_4_ sample. Magnetic field-dependent *R*_*yx*_ at various temperatures. The inset shows the crystal structure of MnBi_2_Te_4_ and an optical image of few-layer flakes of MnBi_2_Te_4_ cleaved by an Al_2_O_3_-assisted exfoliation method. **a** Reproduced with permission from Huang et al., Nature 546, 271 (2017). Copyright 2017 Nature Publishing Group [42]. **b** Reproduced with permission from Gong et al., Nature 546, 265 (2017). Copyright 2017 Nature Publishing Group [43]. **c** Reproduced with permission from Fei et al., Nat. Mater. 17, 778 (2018). Copyright 2018 Nature Publishing Group [44]. **d** Reproduced with permission from Deng et al., Science 367, 895 (2020). Copyright 2020 The American Association for the Advancement of Science [50]

Recently, another member of the 2D vdW family, the 2D magnet, has been obtained experimentally [19, 41]. This breakthrough immediately attracted extensive attention to explore the field of 2D magnetism. Xu’s group first reported that CrI_3_ down to the monolayer exhibits an Ising ferromagnetism with strong out-of-plane magnetic anisotropy by the magneto-optical Kerr effect (MOKE) technique (Fig. 3a) [42]. Moreover, CrI_3_ exhibits a layer-dependent magnetic phase, where monolayer and trilayer CrI_3_ are ferromagnetic while the bilayer is antiferromagnetic. Gong et al. reported another 2D material, Cr_2_Ge_2_Te_6_, which has intrinsic long-range ferromagnetic order in atomic layers [43]. Different from CrI_3_, Cr_2_Ge_2_Te_6_ is reported to be a 2D Heisenberg ferromagnet with small magnetic anisotropy. As shown in Fig. 3b, the ferromagnetic transition temperature of Cr_2_Ge_2_Te_6_ is related to the number of layers. Another popular 2D ferromagnet is Fe_3_GeTe_2_, which is a vdW ferromagnetic metal composed of layered Fe/FeGe/Fe, sandwiched between two Te atom layers [44]. The anomalous Hall effect has been used to study magnetism of Fe_3_GeTe_2_, and the results show Fe_3_GeTe_2_ has strong magnetic anisotropy with an easy magnetization direction parallel to the c-axis and a Curie temperature of 230 K (Fig. 3c) [45]. However, the Curie temperature of these materials is lower than room temperature, which is a big obstacle for devices application. Having Curie temperature above room temperature is a prerequisite for the practical application of two-dimensional magnetic materials. Researchers have prepared room temperature ferromagnetic monolayers 1 T-VSe_2_ by molecular beam epitaxy (MBE) [41]. The recently reported few-layer 1 T-CrTe_2_ exhibited the Curie temperature as high as 316 K [46], which provides the possibility for the application of 2D spintronic devices in the future. In addition to 2D ferromagnetic materials, 2D antiferromagnetic materials are widely reported, such as FePS_3_ [47], MnPS_3_ [48], and CrCl_3_ [49]. More surprisingly, the team of Zhang Yuanbo recently reported magnetic field-induced QAHE in an intrinsic magnetic topological insulator MnBi_2_Te_4_ [50]. MnBi_2_Te_4_ is an antiferromagnet with intralayer ferromagnetism and interlayer antiferromagnetism. By probing quantum transport, an exact quantization of the anomalous Hall effect in a pristine five-layer MnBi_2_Te_4_ flake was observed at a moderate magnetic field of above *μ*_0_*H* ~ 6 T at low temperature (Fig. 3d).

## Elementary Functionalities of 2D Spintronic Device Operations

Recent developments in emerging 2D materials and some advanced characterization techniques have allowed the field of 2D spintronics to develop rapidly [51–53]. The key issues for the realization of spintronic devices include spin-charge conversion, spin transport, and spin manipulation. The efficient generation and detection of spin current is the major challenge to developing 2D spintronic devices that replace the electrical ones. Spin transport desires a suitable transport channel with long spin lifetime and long-distance spin propagation. Spin manipulation is required to control the spin current and achieve device functionality.

### Spin–Charge Conversion

Many methods are proposed to achieve spin-to-charge conversion, such as by electrical spin injection/detection or by utilizing the spin Hall effect and Edelstein effects, which originate from the SOC [54–56]. However, the spin Hall effect usually occurs in bulk materials, while the Edelstein effect is usually considered as an interface effect [55].

**Fig. 4:**
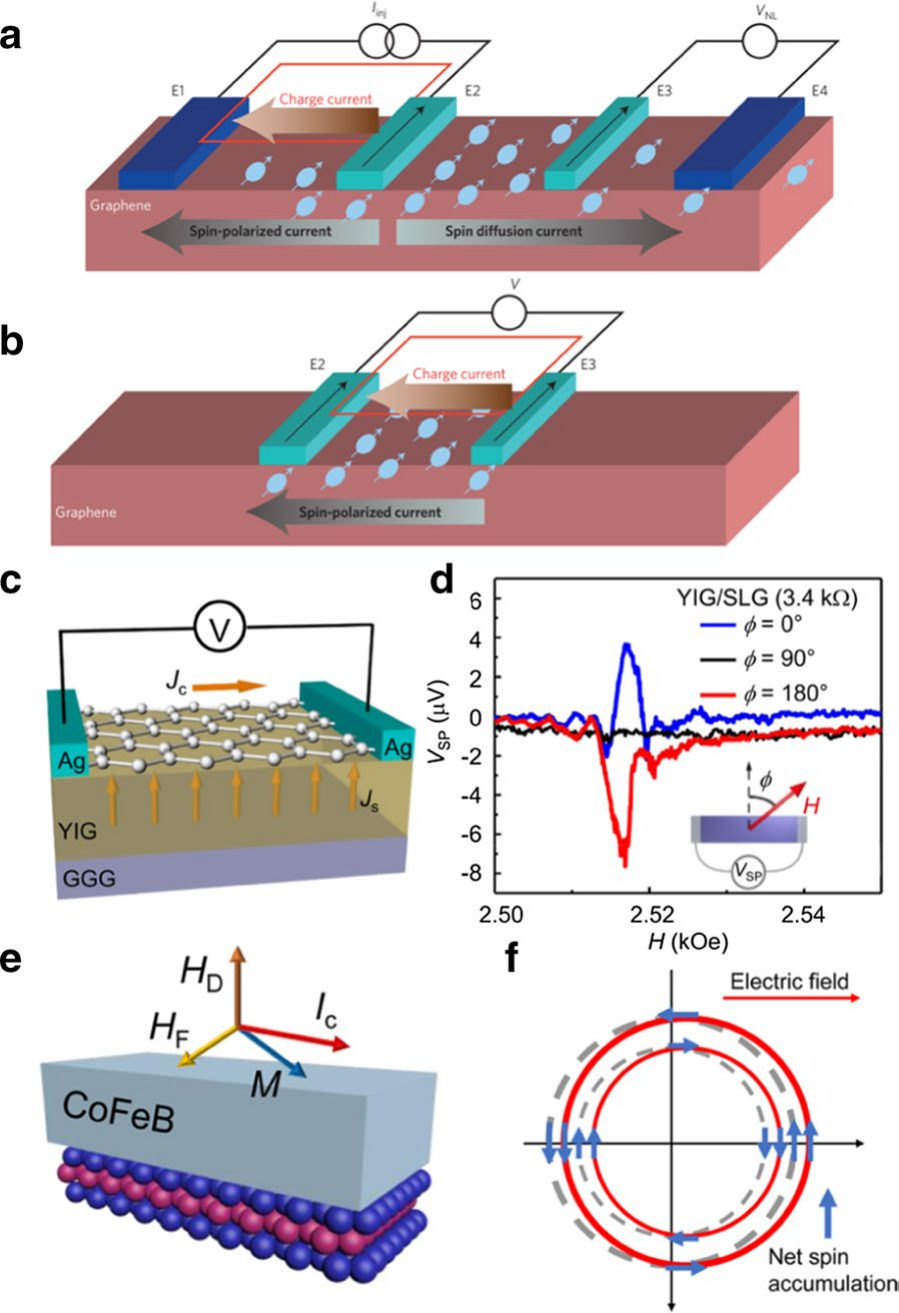
Spin and charge conversion in 2D materials. **a** Electrical spin injection and detection with nonlocal measurement geometries. **b** Electrical spin injection and detection with local measurement geometries. **c** Spin-to-charge conversion in graphene on YIG, a ferromagnetic insulator. The spin current is generated from spin pumping from YIG and is converted to charge current in the graphene. **d** Magnetic field dependence of the spin pumping voltage measured on YIG/Graphene **e** SOT measurements for the MX_2_/CoFeB bilayer. The MX_2_ represents MoS_2_ and WSe_2_. **f** The illustration of induced spin accumulation by the Rashba–Edelstein effect (REE) at the interface of MX_2_/CoFeB under an external electric field. **a**, **b** Reproduced with permission from Han et al., Nat. Nanotechnol. 9, 794 (2014). Copyright 2018 Nature Publishing Group [14]. **c**, **d** Reproduced with permission from Mendes et al., Phys. Rev. Lett. 115, 226601 (2015). Copyright 2015 American Chemical Society [68]. **e**, **f** Reproduced with permission from Shao et al., Nano Lett. 16, 7514 (2016). Copyright 2016 American Chemical Society [71]

The “nonlocal” and “local” measurements are commonly utilized to perform electrical spin injection/detection into a channel material [14]. For nonlocal measurement (Fig. 4a), electrode E2 is a ferromagnetic metal as a spin injector, and E3 is a ferromagnetic electrode as a spin detector. An applied current flows from electrodes E1 to E2, and E3 and E4 are used to detect the diffused pure spin current signal. The polarity of the measured voltage between E3 and E4 depends on the magnetization configurations of electrode E2 and E3. This method can gain a pure spin current without charge current, while the “local” measurements get a mixed signal of spin current and charge current (Fig. 4b). The difference of voltage between the parallel and antiparallel magnetization alignments of the electrodes E2 and E3 is considered as the signal of spin transport.

Hill et al. first reported the injection of spin into graphene by using soft magnetic NiFe electrodes [57]. However, the spin injection efficiency is estimated to be relatively low, around 10%, which could be attributed to the conductance mismatch between ferromagnetic metal and graphene. Then, some researchers proposed using an insulating barrier such as Al_2_O_3_ or MgO as a layer to tune interfacial spin-dependent resistivity and enhance the spin injection efficiency [58–60], but growing a high-quality layer of oxide is a major challenge. Some methods have been used to improve the oxide layer growth technique or change to another interfacial oxide layer, such as a layer of TiO_2_ or HfO_2_ [61, 62]. However, interfacial spin-dependent resistivity is still the fundamental problem, which leads to a low spin injection efficiency. One 2D insulation material, hexagonal boron nitride (h-BN), has a crystal structure similar to that of graphene. Theoretical and experimental studies have shown that using h-BN as a tunnel barrier can produce a high-quality interface and greatly improve the spin-injection efficiency of graphene. Few-layer h-BN exhibits better spin injection performance than monolayer h-BN [63, 64]. Nevertheless, these research results still leave a big gap to be filled before practical application is possible. Ultimately, to achieve perfect (100%) spin injection requires much research, and 2D materials provide a promising direction, such as 2D heterostructures composed of 2D ferromagnetic materials, 2D tunnel barriers, and 2D transport channels.

The (inverse) Rashba–Edelstein effect is an interface effect originating from the strong SOC, which can be utilized to achieve spin-charge conversion [65]. Although intrinsic graphene has a rather weak SOC, it can achieve efficient spin-charge conversion by using the strong SOC of adjacent material via proximity effect [66, 67]. As shown in Fig. 4c, when graphene is adjacent to the ferromagnetic insulator YIG, the spin current is generated in the YIG layer via spin pumping, then converted to a charge current in graphene by the inverse Edelstein effect [68]. Figure 4d shows the spin pumping voltage curves as a function of the field in the YIG/graphene device. The spin pumping voltages can be detected in the magnetic field perpendicular to the graphene channel. Moreover, when the external magnetic field is turned along the graphene channel, there is no spin pumping voltage. Furthermore, an ionic liquid gating applied on the graphene surface can obviously modulate the properties of graphene to change the spin-to-charge conversion efficiency of YIG/graphene [56].

Unlike graphene, TMDCs with strong SOC are considered to be promising materials for achieving spin-charge conversion [69, 70]. A large spin–orbit torque (SOT) in monolayer TMDC (MoS_2_ or WSe_2_)/CoFeB bilayer structure was generated via current-induced spin accumulation caused by the Rashba–Edelstein effect (Fig. 4e, f) [71]. The field-like torque and damping-like torque were determined via a second-harmonic measurement, and the results show that large-area monolayer TMDCs have potential applications because of their high efficiency for magnetization reversal. In addition, the technique of spin-torque ferromagnetic resonance (ST-FMR) has been used to investigate the spin and charge conversion in TMDCs. For example, an interesting ST-FMR result shows the SOT can be controlled through the crystal symmetry of WTe_2_ in WTe_2_/ Permalloy bilayers. When current is applied along the low-symmetry axis of WTe_2_, an out-of-plane anti-damping torque can be generated [72]. The spin-momentum locking property in TI surface states is useful to achieve spin current injection into adjacent materials via SOT. Due to the strong correlation between the spin polarization direction and charge current direction, the spin direction can be manipulated by the charge current in the TIs. Different measurement techniques have been used to investigate the spin-charge conversion, including second harmonic measurement, spin pumping, and ST-FMR. These measurement results demonstrate that it is possible to generate efficient SOT in 2D materials such as TMDCs and TIs.

### Spin Transport

**Fig. 5:**
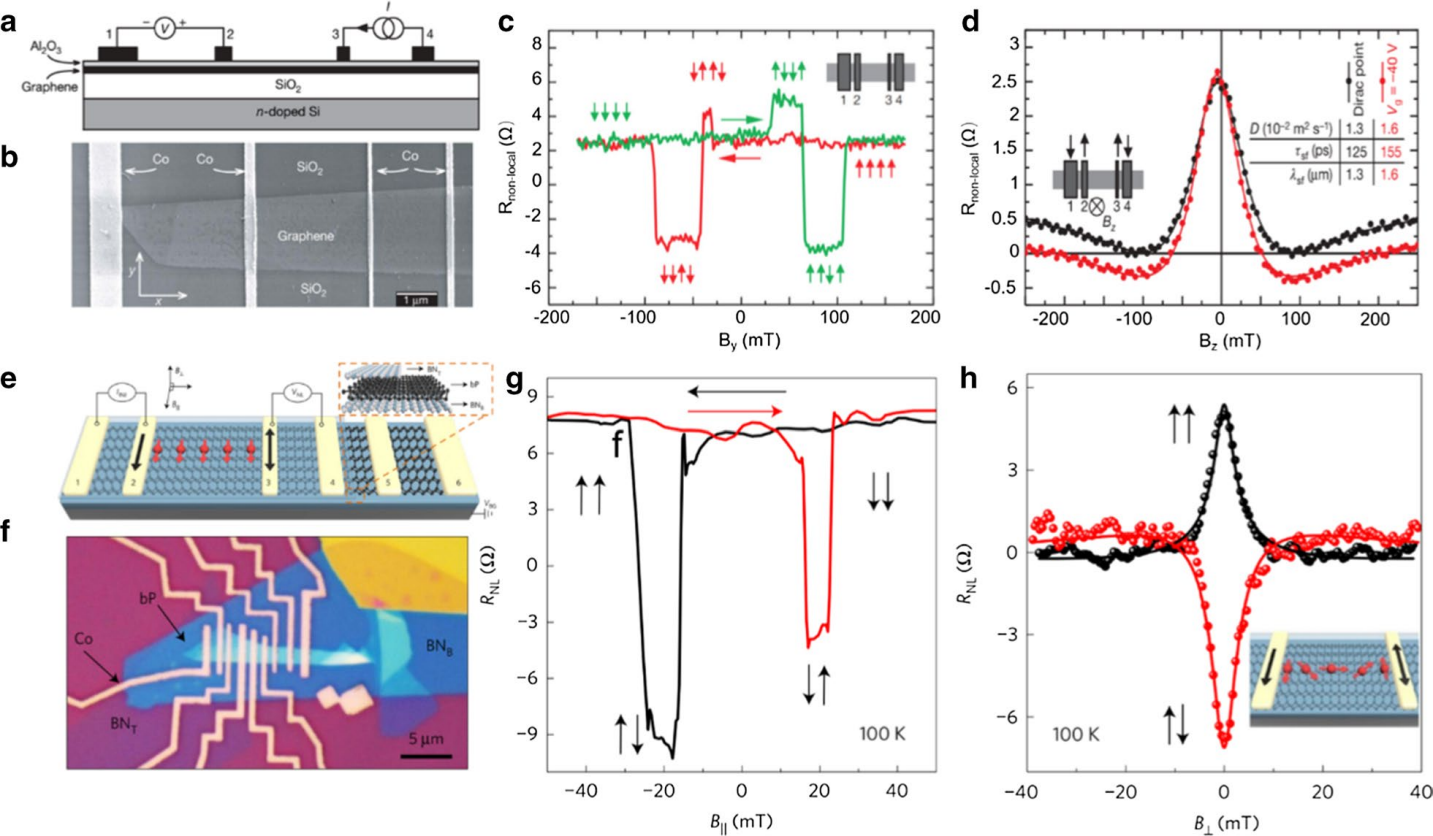
Spin transport in lateral spin valves. **a** Nonlocal spin transport measurement geometries. A current is injected from electrode 3 through the Al_2_O_3_ barrier into graphene and is extracted at contact 4. **b** Scanning electron micrograph of a four-terminal spin valve with single-layer graphene as spin transport channels and Co as four ferromagnetic electrodes. **c** Nonlocal spin valve signal at 4.2 K. The magnetic configurations of the electrodes are illustrated for both sweep directions. **d** Hanle spin precession in the nonlocal geometry, measured as a function of the perpendicular magnetic field *B*_z_ for parallel configurations. **e** Schematics of a black phosphorus spin valve. The inset shows the schematics of the heterostructure. **f** Optical image of the device. **g** Nonlocal spin valve signal as a function of the in-plane magnetic field. The relative magnetization of the injector and detector electrodes are illustrated by vertical arrows, and the horizontal arrows represent the sweeping directions of the magnetic field. **h** Hanle spin precession in the nonlocal geometry, measured as a function of the perpendicular magnetic field *B*_z_ for parallel and antiparallel configurations. The inset shows the spin precession under the applied magnetic field. **a**–**d** Reproduced with permission from Tombros et al., Nature 448, 571 (2007). Copyright 2007 Nature Publishing Group [74]. **e**–**h** Reproduced with permission from Avser et al., Nat. Phys. 13, 888 (2017). Copyright 2017 Nature Publishing Group [84]

The key to spin transport is to get a favorable spin transport channel with a long spin diffusion length and spin relaxation time. Spin relaxation is caused by momentum scattering, so graphene with weak SOC is regarded as an ideal material for spin transport [14, 73]. Tombros et al. [74] realized electronic spin transport and spin precession in a lateral single graphene spin valve at room temperature by nonlocal measurement in 2007. As shown in Fig. 5a, b, the nonlocal spin valve is composed of four-terminal ferromagnetic cobalt as electrodes, a thin Al_2_O_3_ oxide layer as a barrier, and a graphene sheet as the spin transport channel. The measurement signal in Fig. 5c shows that if the ferromagnetic electrodes for spin injection and spin detection have parallel magnetizations, the nonlocal resistance measured by contacts 1 and 2 has a positive value. If the ferromagnetic electrodes for spin injection and spin detection have antiparallel magnetizations, then the nonlocal resistance shows a negative value. The Hanle spin precession can be used to determine spin diffusion length and spin lifetime. As shown in Fig. 5d, the spin lifetime (*τ*_sf_) and spin relaxation length (*λ*_sf_) are 125 ps and 1.3 μm, respectively, in a lateral single graphene spin valve at room temperature. Furthermore, the gate can be used to enhance the spin relaxation length and spin life [75, 76]. Theory predicted that the spin lifetime in pristine graphene can reach 1 μs, whereas the reported experiment values range from picoseconds to a few nanoseconds.

Many improved methods are used to increase spin diffusion length and spin life, and some devices already exhibit long spin diffusion lengths in the micrometer range [13, 77, 78]. For example, graphene epitaxially grown on SiC has high mobility, exhibiting spin transport efficiency up to 75% and spin diffusion length exceeding 100 µm [79]. The h-BN/graphene/h-BN heterostructure exhibits a long-distance spin transport performance, where the spin diffusion length can reach 30.5 μm at room temperature [13]. Spin transport in 2D materials can be affected by diffusion/drift, which can be modulated by applying an electrical field. Ingla-Aynés et al. [80] reported a spin relaxation length up to 90 μm in h-BN encapsulated bilayer graphene by using carrier drift. However, the weak SOC and zero bandgap in intrinsic graphene restrict its prospects for semiconducting spin devices. Black phosphorus has a sizeable direct bandgap and room-temperature mobility of 1000 cm^2^ V^−1^ s^−1^, which make it an ideal semiconducting spintronic material [81–83]. Avsar et al. [84] constructed a lateral spin valve based on an ultrathin black phosphorus sheet and measured its spin transport properties at room temperature via the nonlocal geometry (Fig. 5e, f). The electronic spin transport in Fig. 5g shows that as the magnetization directions of the ferromagnets switch, the nonlocal resistance has a change of Δ*R* ≈ 15Ω. In addition to this, the Hanle spin precession shows spin relaxation times up to 4 ns and spin relaxation lengths exceeding 6 µm (Fig. 5h). The spin transport in black phosphorus is closely related to the charge carrier concentration, so the spin signal can be controlled by applying an electric field.

### Spin Manipulation

Realizing the manipulation of spin is the key to effective device functionalization. Applying a gate voltage can control the carrier concentration in the material, which can be used to manipulate the spin signals [85, 86]. Various 2D materials as spin transport channels have been investigated to realize the adjustment of the spin transport parameters via applying a gate voltage. For example, bias induced graphene can get a spin-injection and detection polarization up to 100% in ferromagnet/bilayer h-BN/graphene/h-BN heterostructure [64]. A gate-tunable spin valve based on black phosphorus can reach a spin relaxation time in the nanosecond range and a long spin relaxation length [84]. For a semiconducting MoS_2_ channel, applying a gate voltage can still get a relatively long spin-diffusion length, larger than 200 nm [70]. However, a suitable spin field-effect device requires a clear switching ratio, which is a challenge for graphene and even for semiconducting 2D materials [87, 88].

**Fig. 6:**
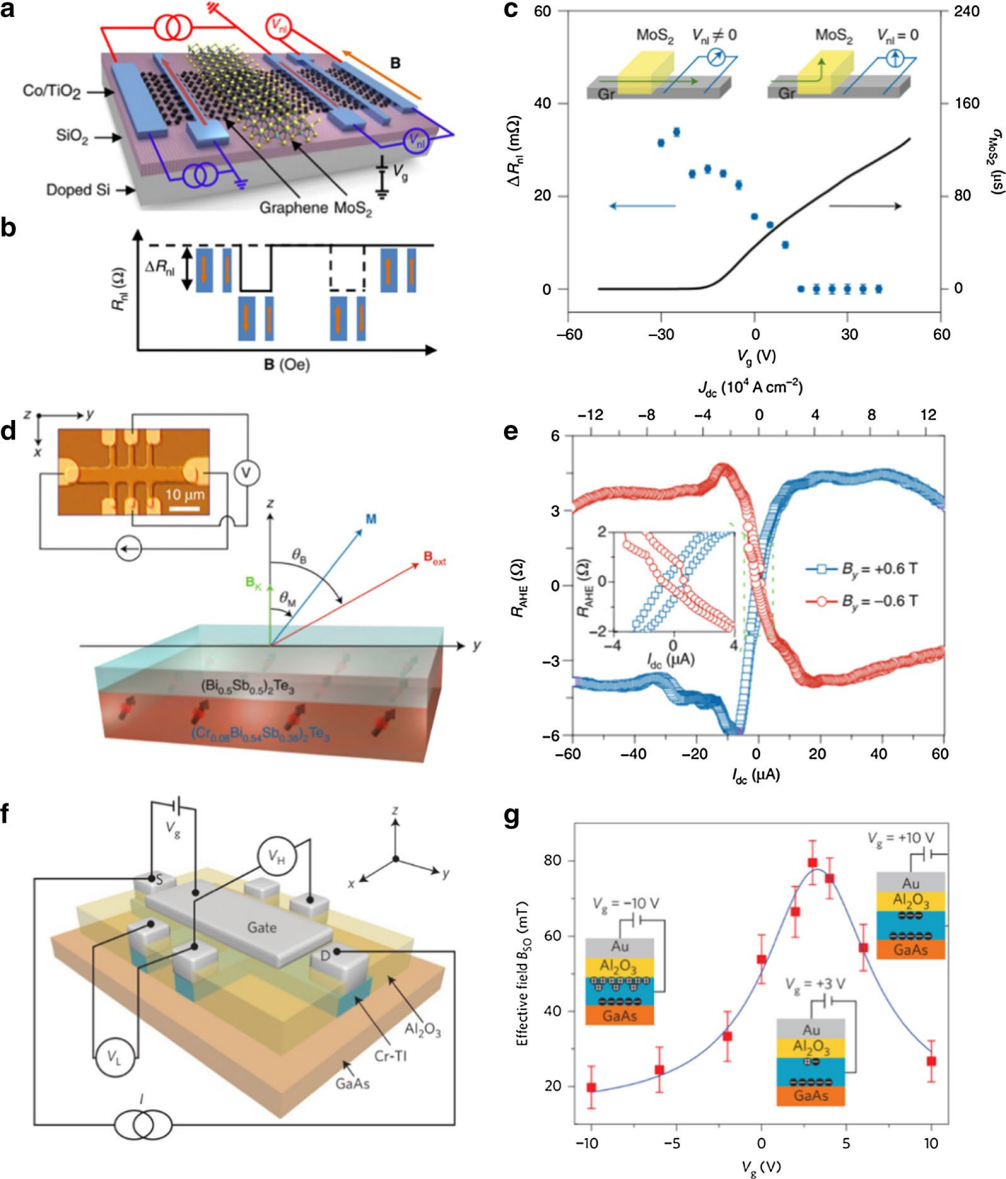
Spin manipulation. **a** Schematic illustration of a 2D spin field-effect switch based on a vdW heterostructure of graphene/MoS_2_ with a typical nonlocal magnetoresistance measurement. **b** The nonlocal resistance *R*_nl_ switches between *R*_P_ and *R*_AP_ for parallel and antiparallel magnetization orientations of the Co electrodes. The spin signal is calculated as Δ*R*_nl_ = *R*_P _− *R*_AP_. **c** The plot with blue circles shows the gate modulation of the spin signal Δ*R*_nl_. The solid black line represents the sheet conductivity of the MoS_2_ as a function of *V*_g_. The insets show the spin current path in the OFF and ON states of MoS_2_. **d** Schematic illustration of SOT-induced magnetization switching in a Cr-doped TI bilayer heterostructure. The inset shows illustrations of the Hall bar device and the measurement setting. **e** Experimental results of SOT-induced magnetization switching by an in-plane direct current at 1.9 K while applying a constant in-plane external magnetic field *B*_y_ during the measurement. The inset shows an enlarged version of the circled part in the figure. **f** 3D schematic of the Hall bar structure of the Al_2_O_3_/Cr-TI/GaAs stack with a top Au gate electrode. A gate voltage of *V*_g_ can be applied between the top gate and the source contact. **b** Effective *B*_SO_ as a function of *V*_g_. The inset shows the surface carrier distribution in the Cr-TI layer under *V*_g_ = − 10 V, + 3 V, and + 10 V. **a**–**c** Reproduced with permission from Yan et al., Nat. Commun. 7, 1 (2016). Copyright 2016 Nature Publishing Group [89]. **d**, **e** Reproduced with permission from Fan et al., Nat. Mater. 13, 699 (2014). Copyright 2014 Nature Publishing Group [95]. **f**, **g** Reproduced with permission from Fan et al., Nat. Nanotechnol. 11, 352 (2016). Copyright 2016 Nature Publishing Group [96]

To solve this issue, a vdW heterostructure based on atomically thin graphene and semiconducting MoS_2_ has been developed to achieve a spin field-effect switch via applying a gate voltage (Fig. 6a) [89]. In this structure, the superior spin transport properties of graphene and the strong SOC of MoS_2_ are combined. The applied gate voltage can change the conductivity of MoS_2_ and spin absorption during the spin transport, which results in switching of the spin current between ON and OFF states in the graphene channel (Fig. 6b). Another research effort produced a similar report about the graphene/MoS_2_ vdW heterostructure. In this report, an electric gate control of the spin current and spin lifetime in the graphene/MoS_2_ heterostructure was achieved at room temperature [90]. Moreover, that report pointed out that the mechanism of gate tunable spin parameters stemmed from gate tuning of the Schottky barrier at the MoS_2_/graphene interface and MoS_2_ channel conductivity.

Current-induced SOT is regarded as another efficient strategy to manipulate spin. The spin current, generated by the spin Hall effect within the heavy metals or the Rashba effect at the interfaces, can exert a spin torque to ferromagnets and thereby realize magnetization switching [91–93]. Efficient current-induced magnetization switching via SOT may lead to innovative spintronic applications. Due to strong SOC and time-inversion symmetry breaking, magnetically doped TIs are being considered as a promising material to manipulate spin signals via SOT [94]. Wang’s group first experimentally demonstrated a magnetization switching induced by an in-plane current in an epitaxial Cr-doped TI (Bi_0.5_Sb_0._5)_2_Te_3_/(Cr_0.08_Bi_0.54_Sb_0.38_)_2_Te_3_ bilayer film (Fig. 6c) [95]. The spin Hall angle in the Cr-doped TI film, ranging from 140 to 425, is almost three orders of magnitude larger than that in heavy metal/ferromagnetic heterostructures, and the critical switching current density is below 8.9 × 104 A cm^−2^ at 1.9 K (Fig. 6d). Furthermore, this team also reported an effective electric field control of SOT in a Cr-doped (Bi_0.5_Sb_0.42_)_2_Te_3_ thin film epitaxially grown on GaAs substrate (Fig. 6e) [96]. The gate effect on the magnetization switching was investigated by scanning gate voltage under a constant current and an applied in-plane magnetic field in the film (Fig. 6f). The SOT intensity depends strongly on the spin-polarized surface current in the thin film, and it can be modulated within a suitable gate voltage range. The effective electric field control of SOT in the TI-based magnetic structures has potential applications in magnetic memory and logic devices.

In addition, electrical control of emerged 2D magnets has also been investigated. For example, utilizing electric fields or electrostatic doping can achieve the magnetic conversion of bilayer CrI_3_ antiferromagnetic to ferromagnetic [97]. The coercivity and saturation field of few-layer Cr_2_Ge_2_Te_6_ can be modulated via ionic liquid gating [98]. In contrast to magnetic semiconductor, electrostatic doping can be used to control the carrier concentrations of the ferromagnetic metal, and the ferromagnetic transition temperature of Fe_3_GeTe_2_ can be dramatically raised to room temperature via an ionic gate [99]. The emergence and research of 2D magnets provide a new platform for engineering next-generation 2D spintronic devices.

## Applications of 2D Spintronics

2D materials exhibit great potential for the engineering of next-generation 2D spintronic devices. Graphene with high electron/hole mobility, long spin lifetimes, and long diffusion lengths is a promising candidate for a spin channel. Moreover, graphene can gain magnetism by introducing adatoms, or magnetic proximity effect [23, 25]. The carrier density in proximity-induced ferromagnetic graphene can be modulated by gating, allowing to observe Fermi energy dependence of the anomalous Hall effect conductivity. This result can help understand the physical origin of anomalous Hall effect in 2D Dirac fermion systems. Realizing a ferromagnetic surface state in a TI is predicted to allow several prominent phenomena to emerge, such as the interfacial magnetoelectric effect [33], and the electric field-induced image magnetic monopole [34]. However, the current technology of inducing magnetism in TI is confined to low temperatures, which restrict its potential for applications. A key requirement for useful applications is the generation of room temperature ferromagnetism in the TI. The PNR result shows that the Bi_2_Se_3_/EuS bilayer has a ferromagnetic order at the interface, and this topologically enhanced interfacial ferromagnetism can persist up to room temperature [32]. The topological magnetoelectric response in such an engineered TI could allow efficient manipulation of the magnetization dynamics by an electric field, providing an energy-efficient topological control mechanism for future spin-based technologies.

The STT, and tunnel magnetoresistance (TMR) effects offer alternative approaches for write and read-out operations. The STT effect refers to the reorientation of the magnetization of ferromagnetic materials via the transfer of spin angular momenta. Efficient current-induced magnetization switching via SOT may lead to innovative spintronic applications [71, 100]. Due to strong SOC and time-inversion symmetry breaking, magnetically doped TIs are being considered as a promising material to manipulate spin signals via SOT [93]. TMR refers to magnetization-dependent magnetoresistance behavior. A high TMR ratio is the key to achieve spintronic devices with higher sensitivity, lower energy consumption. 2D materials with high-quality crystal and sharp interfaces can achieve some new functionalities such as spin filtering. The 2D vdW MTJ consists of a 2D magnetic CrI_3_ layer as a spin filtering tunnel barrier, which reaches a value of TMR up 19,000% [101]. Progress in the fabrication of graphene-based and other 2D heterostructures has led to the optimization of long-distance spin diffusion (up to tens of micrometres), as well as directional guiding of the spin current [13, 64]. Spin manipulation, electrical gating [56], electrical field induced drift [80], SOT-induced switching [95, 96], and the magnetic proximity effect [25, 32] have been explored to develop next-generation MRAM.

## 2D Spintronic Devices for Memory Storage and Logic Applications

Great efforts have been made to search for new 2D spintronic devices. According to the function, 2D spintronic devices can be classified as memory storage or logic devices. Here we focus on several important 2D spintronic devices, including the 2D magnetic tunnel junction (MTJ), 2D spin field-effect transistor (sFET), and 2D spin logic gate.

### 2D MTJ

The discovery of the GMR opens the door for 2D spintronics. However, TMR has a stronger magnetoresistance ratio than GMR, so TMR holds greater potential in magnetic storage applications. The TMR structure consists of two ferromagnetic layers and an intermediate insulating layer, which is called the MTJ. The tunneling probability is related to the density of states near the Fermi energy in the ferromagnetic layers. When the two magnetic layers are parallel, the similar density of states for each spin-state can provide more available states for tunneling, resulting in a low resistance state. On the other hand, when the layers are antiparallel, a mismatch between spin channels of the source and sink will result in a high resistance state. Some issues in traditional thin-film MTJs limit the achievement of a high TMR ratio, such as the quality of the insulation barrier and the thermal stability [102]. 2D materials with high-quality crystal and sharp interfaces may offer promising routes to address these issues and even achieve some new functionalities such as spin filtering.

Karpan et al. first explored graphene layers as the barrier in vertical MTJ by computational means in 2007 [103]. They proposed a match between the band structure of graphene and that of nickel, predicting a large spin polarization close to 100%, which can result in a large TMR up to 500%. However, the subsequent experimental results show that the MTJs based on graphene exhibit a very low TMR. Compared to monolayer or bilayer graphene, the few-layer MTJ holds the highest recorded TMR signal of up to 31% in graphene-based MTJs [11, 15]. In addition to graphene, some other 2D materials have been explored as tunneling barrier layers, including insulating h-BN and semiconducting TMDCs [104, 105]. Piquemal-Banci et al. [63] fabricated Fe/h-BN/Co junctions where the h-BN monolayer was directly grown on Fe by using the chemical vapor deposition (CVD) method, observing large spin signals of TMR and the spin polarization of *P* ~ 17%. MTJs based on MoS_2_ or WSe_2_ were reported to have only a few percent of the TMR signal; further exploration is needed to achieve a high TMR ratio.

**Fig. 7:**
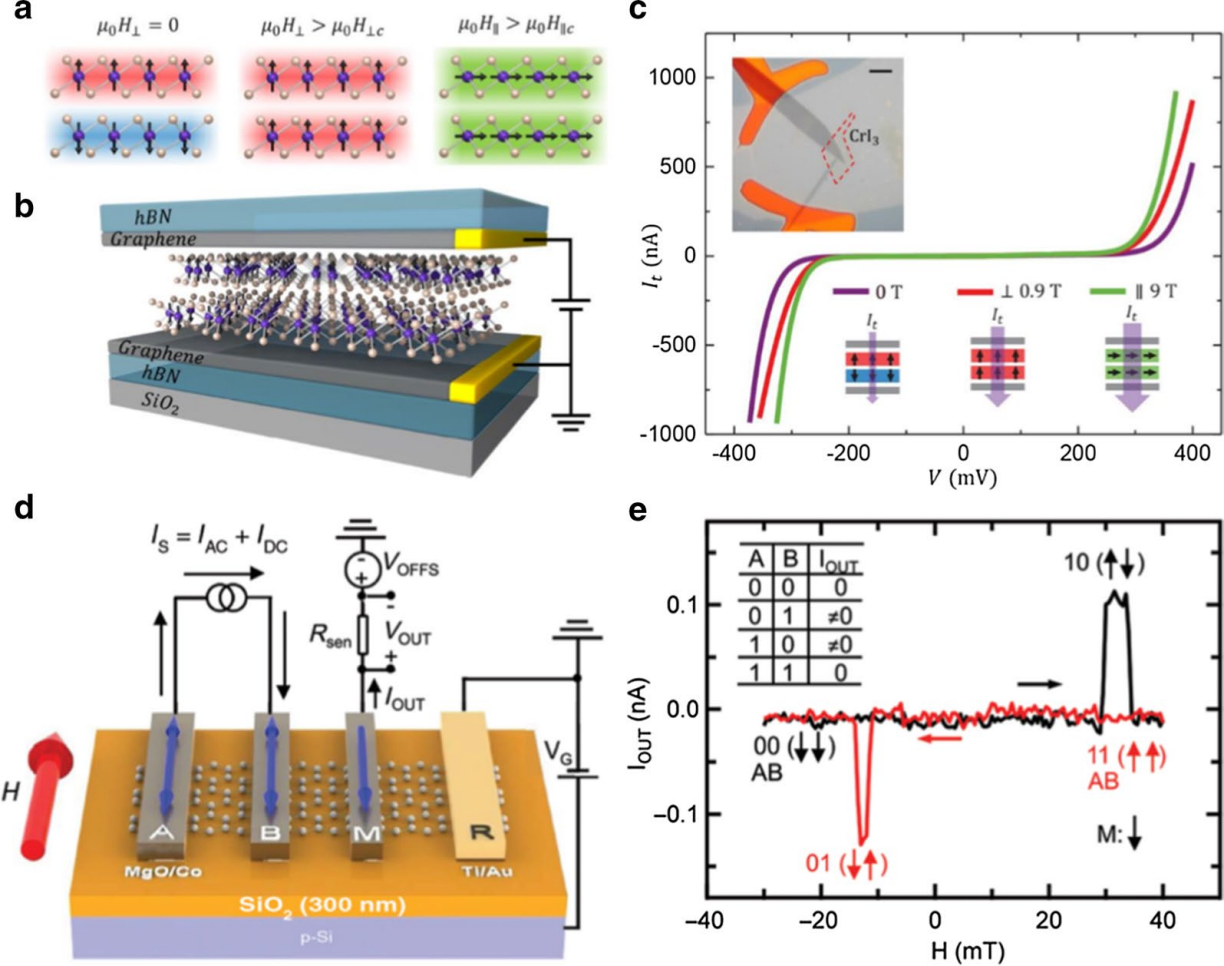
2D spintronic Devices. **a** Magnetic states of bilayer CrI_3_ with different external magnetic fields. **b** Schematic illustration of a 2D spin-filter MTJ with bilayer CrI_3_ sandwiched between graphene contact. **c** Tunneling current of a bilayer CrI_3_ sf-MTJ at selected magnetic fields. The top inset shows an optical image of the device, and the bottom inset shows the schematic of the magnetic configuration in different magnetic fields. **d** Diagram of a proposed 2D XOR spin logic gate, where A, B, and M are ferromagnetic electrodes on top of a spin transport channel. *I*_s_ and *I*_out_ denote the injection and detection currents, respectively. The magnetizations of the electrodes are input logic 1 and 0. The detected current *I*_out_ serves as the logic output. **e** *I*_out_ measured as a function of *H*. Vertical arrows indicate the magnetization states of A and B. The top-left inset shows the table of XOR logic operation. **a**, **b** Reproduced with permission from Song et al., Science (2018). Copyright 2018 The American Association for the Advancement of Science [101]. **c**, **d** Reproduced with permission from Wen et al., Phys. Rev. Appl. 5, 044003 (2016). Copyright 2016 American Chemical Society [118]

Emerging 2D magnetic materials exhibit many surprising properties. When the magnetizations in bilayer CrI_3_ are switched to different magnetic configurations (Fig. 7a), the MTJ based on CrI_3_ exhibits a giant TMR produced by the spin-filtering effect [101, 106, 107]. As demonstrated in Fig. 7b, the 2D vdW MTJ consists of a 2D magnetic CrI_3_ layer as a spin filtering tunnel barrier, graphene as a contact electrode, and h-BN as an encapsulation layer to prevent device degradation. The transport result shows that the TMR is enhanced as the CrI_3_ layer thickness increases, and it reaches a value of 19,000% in four-layer CrI_3_ based MTJ at low temperature (Fig. 7c) [101]. Subsequently, Xu’s group also reported gate-tunable TMR in a dual-gated MTJ structure based on four-layer CrI_3_. The TMR can be modulated from 17,000 to 57,000% by varying the gate voltages in a fixed magnetic field [108, 109]. Moreover, with few-layer Fe_3_GeTe_2_ serving as ferromagnetic electrodes, the TMR in Fe_3_GeTe_2_/h-BN/Fe_3_GeTe_2_ heterostructures can reach 160% at low temperature [110]. More interestingly, Zhou et al. reported a theoretical investigation of a VSe_2_/MoS_2_/VSe_2_ heterojunction, where the VSe_2_ monolayer acts as a room-temperature ferromagnet, and the large TMR can reach 846% at 300 K [111]. On the other hand, the strong spin Hall conductivity of MoS_2_ holds potential to switch the magnetization of the VSe_2_ free layer by SOT. Therefore, they put forward the concept of SOT vdW MTJ with faster reading and writing operations, which offers new opportunities for 2D spintronic devices.

### 2D sFET

Datta and Das first proposed the idea of the sFET in 1990 [112]. The sFET consists of the source and sink ferromagnetic electrodes, and a two-dimensional electron gas (2DEG) channel which can be controlled by an electrical gate. The gate voltage can result in a spin precession and, consequently, a change in the spin polarization of the current on the channel. Since switching the current through the device requires only little energy and a short time, sFET is expected to be a 2D spintronic device with low power consumption and high computing speed.

As mentioned in the previous section, graphene with high carrier concentration and weak SOC is considered to be a promising candidate as a spin transport channel [113]. Michetti et al. [76] designed a double-gate field-effect transistor structure, where bilayer graphene acts as the transport channel. Theoretical analysis shows that the spin precession of carriers in the graphene channel can be turned on and off by the application of a differential gate voltage. Experimentally, Avsar et al. first reported a dual-gated bilayer graphene structure with h-BN as a dielectric layer, where the spin current propagation in bilayer graphene channel can be controlled by exerting a vertical electric field [114]. The transport results show that the spin-relaxation time decreases monotonically as the carrier concentration increases, and the spin signal exhibits a rapid decrease, eventually becoming undetectable close to the charge neutrality point. A suitable spin field-effect device requires a clear switching ratio, which is a challenge for graphene.

To solve this issue, a graphene/MoS_2_ heterostructure has been developed to achieve a spin field-effect switch via applying a gate voltage. Two independent groups demonstrated that the applied gate voltage can change the conductivity of MoS_2_ and result in spin absorption during the spin transport, which gives rise to switching the spin current between ON and OFF states in the graphene channel [89]. Due to the low spin injection efficiency and rapid spin relaxation in channels, it is a challenge to achieve a large high-to-low conductance ratio in 2D sFET device. However, the discovery of 2D magnetic crystals provides new opportunities to explore new 2D spintronic devices. Kin Fai Mak’s group reported a spin tunnel field-effect transistor (sTFET) based on a dual-gated graphene/CrI_3_/graphene heterostructure [115]. By using bilayer CrI_3_ as a magnetic tunnel barrier, the applied gate voltage can switch magnetization configurations of bilayer CrI_3_ from interlayer antiferromagnetic to ferromagnetic states under a constant magnetic field near the spin-flip transition. Distinct from conventional spin transistors, these devices rely on electrically controlling the magnetization configuration switching rather than the signal of spin current in the channel. This technique allows the sTFET devices to achieve a large high–low conductance ratio approaching 400%, which provides a new approach for exploring memory applications.

### 2D Spin Logic Gate

Dery and Sham first reported a spin logic device based on an “exclusive or” (XOR) gate [116]. The XOR logic gate structure includes a semiconductor channel and three ferromagnetic terminals. An XOR logic operation can be implemented by different spin accumulations, which is caused by different magnetization configurations of the input terminals [117]. Experimentally, the proposed three-terminal XOR logic gate achieved logical operations in a graphene spintronic device at room temperature [117–119]. As shown in Fig. 7c, the device includes single-layer graphene as the channel, and three ferromagnetic terminals composed of A, B, and M Co electrodes with MgO tunnel barriers. The magnetization of the electrodes A and B represents the input states 0 or 1, and the current of the electrode M acts as the output state. The magnetizations of input electrodes A and B will be switched by varying an applied external magnetic field, which results in a different spin accumulation in the M electrode, corresponding to a different output current. If the input A and B electrodes have identical contributions to the output M electrode, then the current in the output ferromagnetic terminal has a detectable value only when the magnetization of input ferromagnetic terminals are antiparallel (01 or 10). When the magnetizations of the input ferromagnetic terminals are parallel (00 or 11), the output current is almost zero. Therefore, the XOR logic operation can be achieved (Fig. 7d).

Dery et al. further designed a reconfigurable magnetologic gate with five-terminal structure combining two XOR gates-XOR (A, X) and XOR (B, Y) with a shared output terminal, M [119]. Similar to the three-terminal XOR logic gate, the different magnetic configurations of input electrodes give rise to the different spin accumulation in the output terminal M, which results in different output signals. By analogy, a finite number of these XOR gates can be used to implement any binary logic function. Subsequently, other groups extended this theoretical design to experimental studies by constructing graphene spin logic gates [120–122]. Various modeling, simulation, and experimental explorations of 2D spin logic gates have helped to accelerate the progress toward building practical spin logic applications. However, two key issues remain in the research of graphene spin logic gates. The first one is to balance the contributions of two input terminals to the output one. The other one is to eliminate the influence of background signals on the output.

## Challenges and Opportunities in 2D Spintronics

As discussed above, much theoretical and experimental research has been carried out to explore spintronics based on 2D materials, and considerable progress has been achieved [15, 123, 124]. However, great challenges still need to be addressed for the practical application of 2D memory and logic applications. We now discuss three of these: physical mechanisms, materials science, and device engineering.

### Physical Mechanisms

Due to the complexity of the experiments, the proposed theoretical research and experimental results usually have large discrepancies. For example, based on the mechanism of spin relaxation, theory predicted that the spin lifetime for pristine graphene would be up to 1 μs, whereas experimental values range from tens of picoseconds to a few nanoseconds [14, 57, 103]. Furthermore, the spin injection efficiency of graphene measured experimentally ranges from a few percent to 10%, which is far smaller than the theoretical prediction value of 60–80% [125]. These differences indicate that more in-depth physical mechanisms and accurate theoretical models need to be proposed and developed to better guide the research direction and analyze the experimental results.

### Materials Science

2D materials provide an ideal platform to construct various heterostructures for spintronic applications. However, there are still many major problems in 2D materials. For example, stability is a great challenge for 2D materials. Most 2D materials of thickness close to the atomic level are susceptible to moisture, oxygenation, and temperature, especially the recently emerging 2D magnetic materials, which must be peeled off in a glove box with ultra-low water and oxygen content. Besides this, most currently available 2D magnets rely on mechanical exfoliation, and their low magnetic transition temperature is far below room temperature. These are significant limitations because stability in air, convenient wafer-scale synthesis, and operation above room temperature are prerequisites for 2D materials used in practical applications.

### Device Engineering

Breakthroughs have been made in the fundamental study of 2D spintronics, such as enhanced spin injection efficiency by using 2D tunnel barriers h-BN, long spin diffusion length up to 30 μm at room temperature in graphene-based 2D heterostructures [13], and high TMR up to 19,000% by using 2D magnets as spin filter barriers [101]. Based on the study of 2D spintronic devices, it is promising to develop the low-power device applications, including advanced magnetic memories and spin logic circuits, which are compatible with the existing complementary metal-oxide semiconductor (CMOS) electronics. However, the design and application of functional 2D spintronic devices are still in the early theoretical prediction and proof-of-concept stage.

### Opportunities

2D spintronics is an important scientific research field with many potential applications for future technologies. As mentioned above, considerable challenges currently remain, but there are also many opportunities. Spin valves based on graphene as the spin transport channel can exhibit a long spin diffusion length up to 30 mm at room temperature [13]. Magnetic tunnel junctions with four-layer CrI_3_ as spin filter tunneling barriers show giant TMR up to 19,000% at low temperatures [101]. The magnetic transition temperature of Fe_3_GeTe_2_ can reach above room temperature via an ionic liquid gate or when tailored by a TI [99, 126]. Spin-polarized current can be injected from WTe_2_ into magnetic substrates by SOT switching [127]. New concepts of spin tunneling field-effect transistors based on 2D magnets CrI_3_ have been proposed as well. The demonstration of giant TMR, the efficient voltage control of 2D magnetism, and the magnetization switching in 2D magnets by STT or SOT all open up opportunities for potential next-generation spintronic devices based on atomically thin vdW crystals [21, 100].

## Conclusion

The study about the magnetic properties of 2D materials is of great significance to the development of 2D spintronics. The magnetic interaction in graphene and TIs has scarcely been explored, and recently discovered 2D magnets also provide an ideal platform to study 2D magnetism. Great progress has been made in 2D spintronics in recent decades, especially in graphene spintronics. However, the origin of spin relaxation in graphene is still a major open question, and further improvement in the spin lifetime and spin diffusion length remains an important research direction for graphene-based spintronic devices. The practical application of 2D spintronic devices still requires meeting great challenges, including related physical mechanisms, materials science, and device engineering. The development of technology, the improvement of theoretical models, and the exploration of new materials all provide more opportunities for new-generation 2D spintronic device applications in the future.

## Data Availability

Not applicable.

## Abbreviations

2D: Two-dimensional
GMR: Giant magnetoresistance effect
STT-MRAM: Spin-transfer-torque magnetoresistive random-access memory
vdW: Van der Waals
SOC: Spin–orbit coupling
TMDCs: Transition metal dichalcogenides
TIs: Topological insulators
SQUID: Superconducting quantum interference device
YIG: Yttrium iron garnet
QAHE: Quantum anomalous Hall effect
PNR: Spin-polarized neutron reflectivity
MOKE: Magneto-optical Kerr effect
MBE: Molecular beam epitaxy
h-BN: Hexagonal boron nitride
SOT: Spin–orbit torque
ST-FMR: Spin-torque ferromagnetic resonance
MTJ: Magnetic tunnel junction
sFET: Spin field-effect transistor
TMR: Tunneling magnetoresistance
CVD: Chemical vapor deposition
2DEG: Two-dimensional electron gas
sTFET: Spin tunnel field-effect transistor
XOR: Exclusive or
CMOS: Complementary metal-oxide semiconductor
